# Stabilising graphite anode with quasi-solid-state electrolyte for long-life lithium–sulfur batteries

**DOI:** 10.1557/s43581-025-00139-0

**Published:** 2025-07-14

**Authors:** Zhuangnan Li, Ziwei Jeffrey Yang, Manish Chhowalla

**Affiliations:** Department of Materials Science and Metallurgy, University of Cambridge, Cambridge, CB3 0FS UK

**Keywords:** 2D materials, energy storage, in situ, polymerization, phase transformation

## Abstract

Lithium–sulfur (Li–S) batteries with high energy density are promising for next-generation energy storage but suffer from poor cycle life due to instability of the lithium anode. Commercial graphite anodes used in lithium-ion batteries could be viable for replacement of lithium metal as anode in Li–S batteries. However, graphite anodes are incompatible with the commonly used ether-based electrolytes in Li–S batteries due to the Li^+^ ion and solvent co-intercalation into graphite interlayers, which leads to exfoliation. Here, we report the stabilisation of graphite anode by *in-situ* formation of quasi-solid-state electrolyte (QSSE) in Li–S batteries. The QSSE is formed by the ring-opening of molecular ethers induced by metallic molybdenum disulfide sulfur cathode host to produce long-chain polymers. The resulting gel polymer matrix exhibits ionic conductivity (1.51 mS cm^−1^) comparable to that of liquid electrolyte and provides sufficient redox chemistry for the sulfur cathode without co-intercalation into the graphite anode. Li–S pouch cells based on *in-situ* formed QSSE and graphite anode show a specific capacity of ~ 1200 mAh g^−1^ and 90.3% capacity retention after 200 cycles. Our design addresses the electrolyte limitation associated with graphite anode, enabling graphite to be used in ether-based solvents for safe, cost-effective and stable Li–S batteries.

**Graphical abstract:**

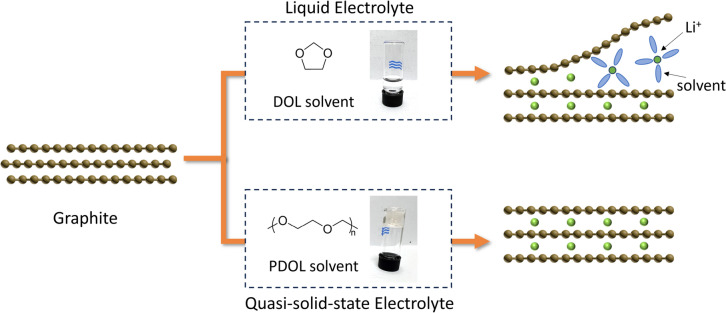

**Supplementary Information:**

The online version contains supplementary material available at 10.1557/s43581-025-00139-0.

## Highlights

- A quasi-solid-state electrolyte was developed to enable stable cycling of commercial graphite anodes in lithium–sulfur batteries.
- The quasi-solid-state electrolyte prevents solvent co-intercalation, enhances safety, and delivers ~ 1200 mAh g−1 with > 90% capacity retention over 200 cycles in pouch cells.

**Supplementary Information:**

The online version contains supplementary material available at 10.1557/s43581-025-00139-0.

## Discussion

- Can the replacement of lithium metal with graphite anode in lithium–sulfur batteries reduce both cost and safety concerns without compromising performance?
- Is quasi-solid-state electrolyte a transitional intermediate or a long-term solution in the broader shift from liquid to solid-state battery technologies?

**Supplementary Information:**

The online version contains supplementary material available at 10.1557/s43581-025-00139-0.

## Introduction

The high theoretical capacity (1672 mAh g^−1^) and earth abundance of elemental sulfur make lithium–sulfur (Li–S) batteries attractive alternatives for energy storage.^1–3^ However, the practical cycle life of Li–S batteries needs to be improved for commercialisation.^1,2^ Li–S batteries consist of sulfur as the cathode and lithium metal as the anode.^3^ The degradation and dendrite growth on lithium anode during cycling along with parasitic reactions between migrated polysulfides and lithium metal limit the battery lifetime.^4,5^ Lithium anode protection strategies such as surface coating, alloying, host design, and electrolyte additives have been widely studied.^5–9^ Moreover, alternative anode materials with better cycling stability have also been considered.^1,10,11^

Graphite has been used as the anode in commercial lithium-ion batteries (LIBs) for more than 30 years because it is stable over many cycles of Li^+^ ion intercalation/deintercalation.^12^ Graphite electrode production is also well established at an industrial scale.^13^ In addition, graphite is less reactive than metallic lithium and thus mitigates some of the safety concerns.^12,13^ However, despite multiple advantages, graphite is found to be unstable in the ether-based electrolyte used in Li–S batteries due to co-intercalation of solvent molecules with Li^+^ ions into the graphite lattice.^14,15^ Such solvent intercalation leads to delamination of graphite anode and rapid cell failure within a few cycles. On the other hand, commercial carbonate electrolytes used in LIBs are not compatible with sulfur cathode because they cause severe side reactions with polysulfide intermediates, resulting in permanent loss of active material.^16^ For these reasons, various strategies have been developed to enable the use of graphite anodes in Li–S batteries, including electrolyte with ultrahigh salt concentration (solvent-in-salt),^17^ and separators that allow the use of different types of electrolytes to be used on the cathode and anode sides.^18^ These approaches remain at the proof-of-concept stage with limited scalability.

**Figure 1:**
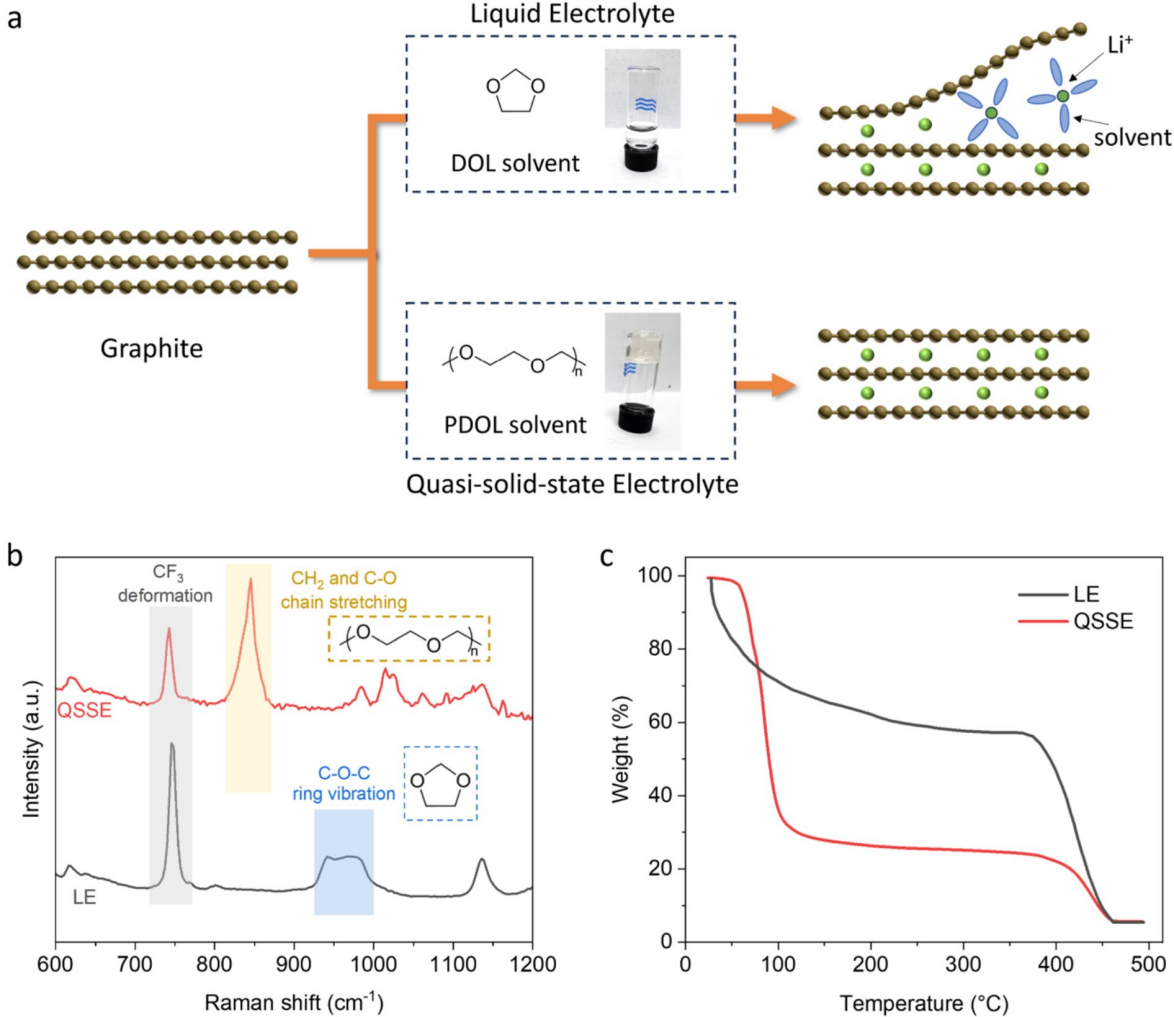
Comparison of structural and thermal properties between LE and QSSE. (a) Schematic illustrating the intercalation of Li^+^ ions into graphite interlayers in different electrolytes, showing that the exfoliation caused by solvent co-intercalation occurs in LE but not in QSSE. (b) Raman spectra with characteristics of ring monomer, chain polymer, and electrolyte salt shaded in blue, yellow, and grey colours, accordingly. (c) Thermogravimetric analyses of LE and QSSE, suggesting improved thermal stability of QSSE prior to DOL boiling point (74 °C). Note that the second weight loss at  ~400 °C is due to the decomposition of LiTFSI.^20^

In this work, we report an ether-based quasi-solid-state electrolyte (QSSE) that allows the stable operation of graphite anode in Li–S batteries. Our previous study has demonstrated that the QSSE can be prepared *in-situ* by triggering the ring-opening polymerisation of 1,3-dioxolane (DOL) with the metallic 1 T phase molybdenum disulfide (1 T MoS_2_) host.^19^ Herein, we further show that, unlike conventional ethers, the resultant polymerised DOL (PDOL) enables the reversible intercalation of Li^+^ ions into the graphite anode without co-intercalation of the solvent (Fig 1a). In addition, the ionic conductivity of the QSSE is similar to that of the liquid electrolyte. The oxidation stability is also improved, extending the electrochemical window by > 1 V. Furthermore, the QSSE exhibits an enhanced flame-retardant property. This design allows the implementation of well-established graphite anode in ether-based electrolyte to realise Li–S batteries with high capacity (~1200 mAh g^−1^) and cycling stability (> 90% retention after 200 cycles).

## Results and discussion

The mechanism of QSSE preparation has been described in our previous study.^19^ Briefly, the sulfur host material in the cathode of our Li–S batteries is the metallic 1 T phase MoS_2_ nanosheets. The Lewis acidity of 1 T MoS_2_ host initiates the reaction. DOL solvents in the conventional liquid electrolyte (LE) undergo a cationic ring-opening polymerisation, forming PDOL and thus the gel polymer QSSE (Figure S1). These structural changes are confirmed by Raman spectroscopy (Fig 1b), where QSSE exhibits C–O chain stretching instead of the C–O–C ring vibration observed in LE. The number average molecular weight of PDOL is  ~3300 g mol^−1^ (DOL monomer is 74 g mol^−1^), suggesting a moderate degree of polymerisation. The as-produced PDOL within the battery cell possesses a long-chain structure with a much larger size than the single cyclic ether molecule of DOL. Therefore, the QSSE solvent is unlikely to co-intercalate with Li^+^ ions into the graphite interlayer, avoiding the exfoliation of graphite anode. In addition, unlike LE that loses weight even at room temperature due to the low boiling point of DOL (~74 °C), QSSE is less volatile and has better thermal stability as measured by thermogravimetric analyses (Fig 1c).

**Figure 2:**
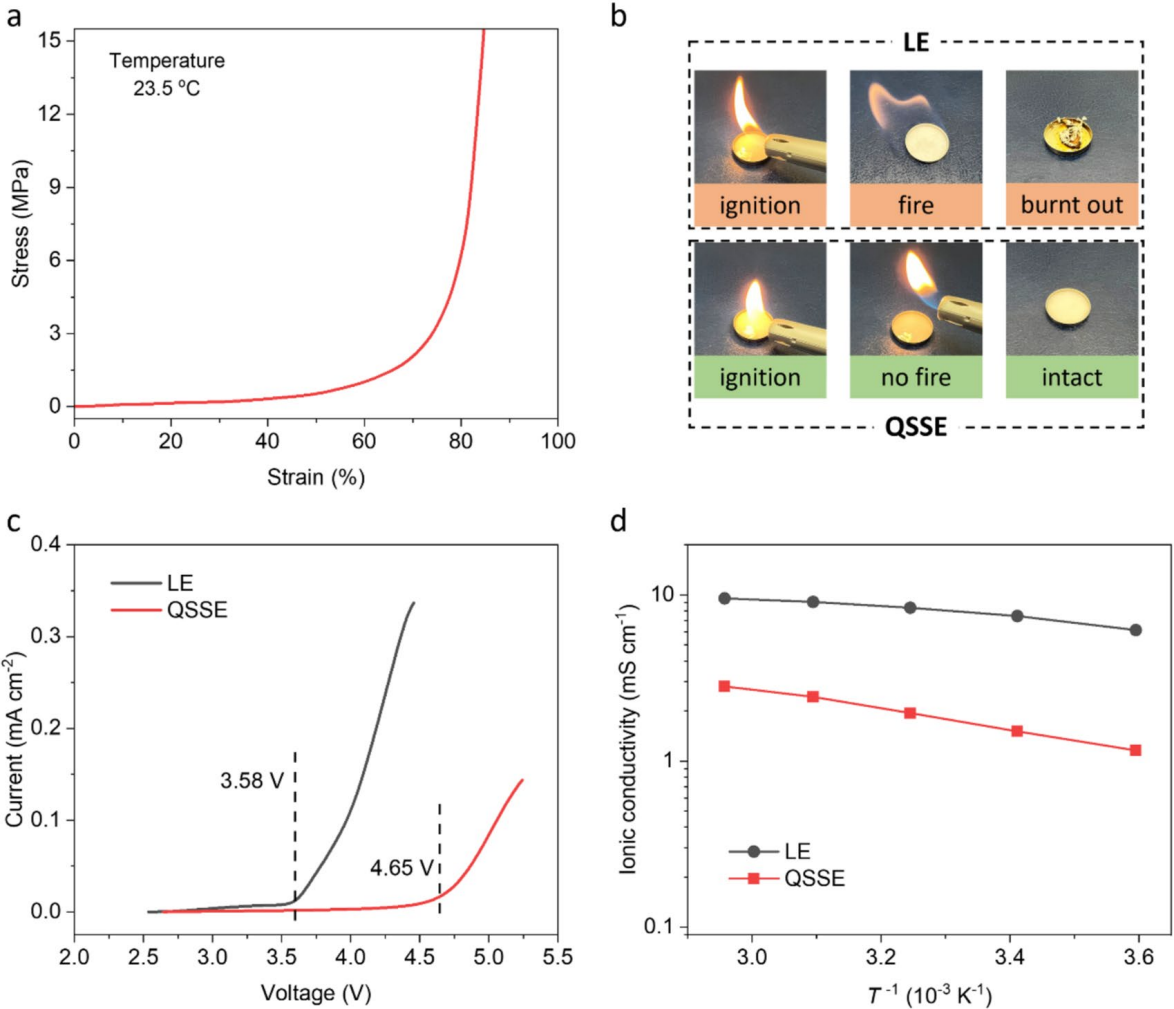
Characterizations of mechanical, safety, and electrochemical properties for QSSE. (a) Uniaxial tensile stress–strain curve of QSSE describing an elastic behaviour. (b) Photographs of LE and QSSE under the combustion test, showing the flame-retardant properties of QSSE. (c) Linear sweep voltammetry of LE and QSSE at a sweep rate of 1 mV s^−1^, demonstrating a better oxidation stability of QSSE. (d) Comparison of ionic conductivity between LE and QSSE under various temperatures.

The stress versus strain curve of the QSSE gel at room temperature shows a typical elastic polymer behaviour of PDOL (Fig 2a). Such elastomer-like mechanical behaviour and strength of QSSE are important for the Li–S battery because the sulfur cathode undergoes  ~80% volume expansion during charge–discharge cycles.^21^ Another crucial feature of QSSE is the flame-retardant property. As shown in Fig 2b, the conventional LE is highly flammable and therefore rapidly ignites and burns. In contrast, QSSE exhibits considerable tolerance to flame exposure and does not catch fire during the combustion test. Both mechanical and thermal stabilities are promising for the use of this electrolyte in practical systems.

In addition to the co-intercalation challenge, conventional DOL-based LE is also not commonly employed in batteries that use intercalating cathodes due to its poor oxidation stability.^22^ Linear sweep voltammetry curves in Fig 2c show that LE starts to decompose at potentials above 3.58 V (*vs*. Li^+^/Li), which is incompatible with commercial cathodes (*e.g.* lithium nickel manganese cobalt oxides usually operate between 3.0–4.2 V). In contrast, QSSE exhibits much better electrochemical stability without noticeable oxidation up to 4.5 V, an extension of voltage window by  ~1 V. In addition to a wider operating voltage, QSSE also possesses ionic conductivity comparable to that of LE. Figure 2d summarises the temperature-dependent ionic conductivity. It is noteworthy that the ionic conductivity of QSSE fits well with Arrhenius or Vogel–Fulcher–Tammann models, suggesting that the temperature range of measurements (5 to 65 °C) is beyond its glass transition temperature. At room temperature, QSSE exhibits ionic conductivity of ~ 1.5 mS cm^−1^, higher than typical values reported for gel polymer electrolytes under similar conditions (< 1 mS cm^−1^).^23^ We attribute this to the *in-situ* formation of QSSE, which leads to superior electrode–electrolyte interface and thereby low interfacial resistance.

**Figure 3:**
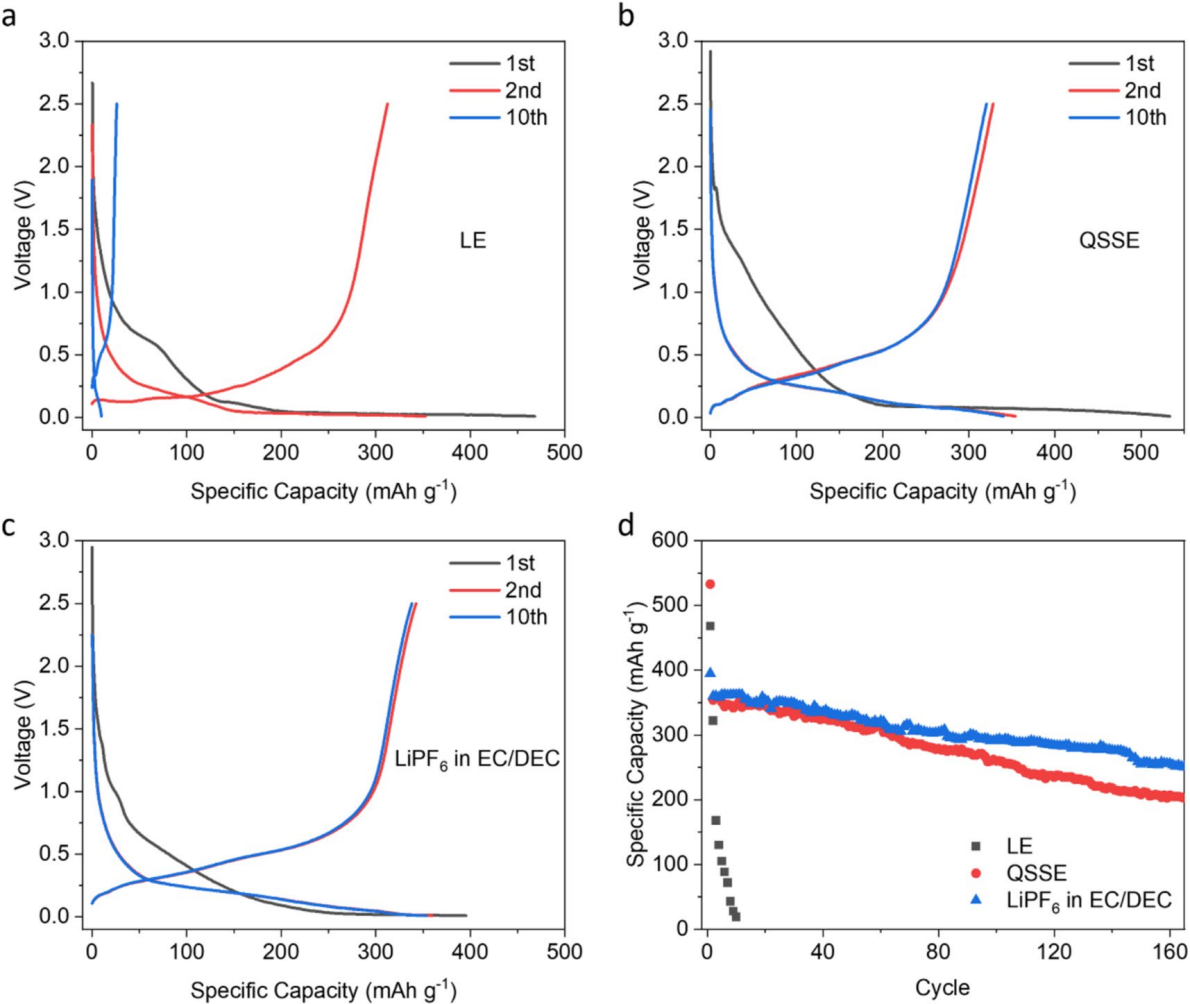
Electrochemical characterizations of Li||graphite cells in different electrolytes. (a-c) Galvanostatic charge–discharge curves at a current density of 0.1 C for Li||graphite cells in LE (a), QSSE (b), and carbonate-based electrolyte (c). (d) Cycling stability of Li||graphite cells in different electrolytes, showing that graphite operates stably in QSSE.

The stability of graphite anode in different electrolytes was studied by galvanostatic charge–discharge (GCD) measurements of Li||graphite cells (Fig 3). GCD profiles indicate that graphite in LE-based cells exhibits a specific capacity of 353 mAh g^−1^ after 1 st cycle initialisation (Fig 3a)—close to its theoretical capacity (372 mAh g^−1^).^12^ However, the subsequent cycles show severe capacity decay with a retention of only 26 mAh g^−1^ after 10 cycles. This is consistent with the literature and is attributed to the exfoliation of graphite as a consequence of solvent co-intercalation. In contrast, while delivering comparable capacity (355 mAh g^−1^ at 2nd cycle) to the cells using LE, graphite in QSSE shows much better reversibility (340 mAh g^−1^ at 10th cycle) (Fig 3b). It can also be seen that both specific capacity and charge–discharge behaviours of graphite in QSSE are similar to those of typical carbonate-based electrolyte (1 M LiPF_6_ in EC/DEC, Fig 3c), suggesting sufficient compatibility of QSSE with graphite. The stable operation of graphite anode in QSSE has been further confirmed over long-term cycling. As shown in Fig 3d, rapid failure of the cell with conventional LE is observed within 10 cycles, whereas the cell using QSSE remains functional for more than 150 cycles.

**Figure 4:**
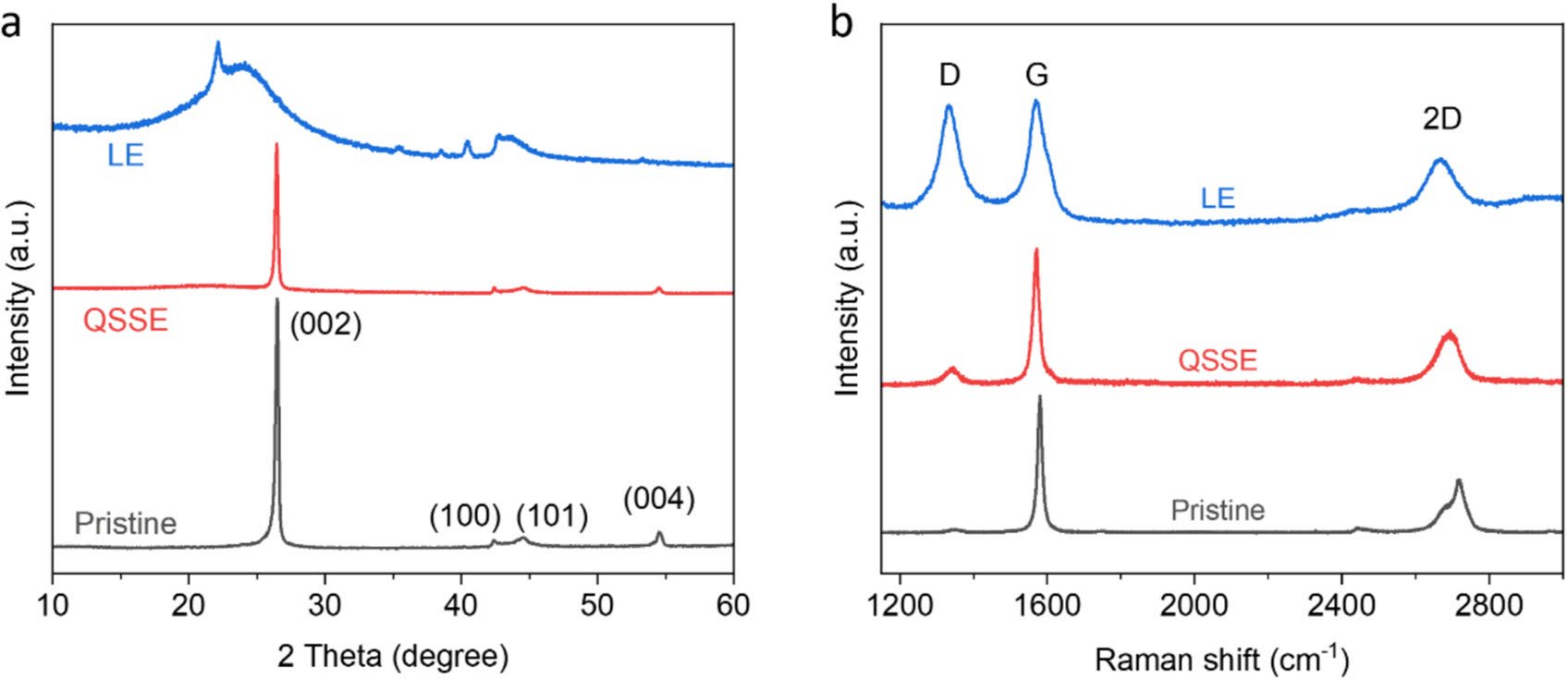
Post-cycling structural characterizations of graphite anodes in different electrolytes. (a, b) XRD patterns (a) and Raman spectra (b) of graphite anode before and after cycling in different electrolytes, demonstrating that the lattice structure is destroyed in LE whereas maintained in QSSE.

To investigate the structure of graphite anode and provide understanding of its stability in QSSE, X-ray diffraction (XRD) was used to characterise the electrodes after cycling. For pristine electrode without cycling, an intense peak at 26.5° is observed on the XRD pattern, corresponding to the (002) plane of graphite (Fig 4a). For the graphite cycled in conventional LE, this sharp peak disappeared, indicating that the original graphite lattice is destroyed by the co-intercalation of DOL solvents. In comparison, such a signature peak is still present after GCD cycles in QSSE, suggesting that the graphite is well maintained without exfoliation, in good agreement with its cycling stability (Fig 3d). Raman spectra also confirm that the graphitic structure (as indicated by G band) is retained after cycling in QSSE, whereas considerable defects and disorder (as indicated by the appearance of prominent D band) are observed for the graphite anode cycled in LE (Fig 4b).

**Figure 5:**
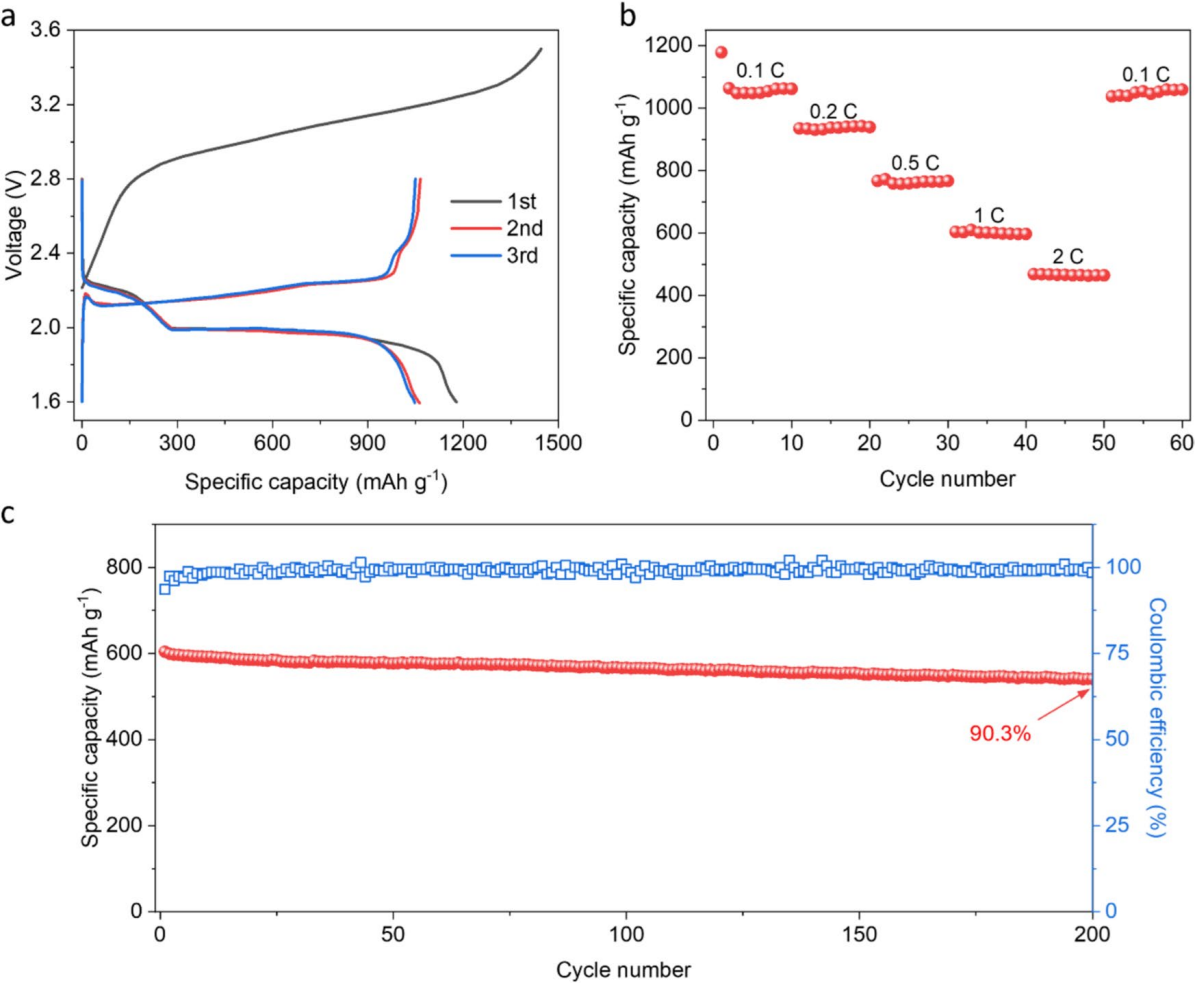
Fabrication of QSSE-based Li–S batteries with graphite anode. (a) Galvanostatic charge− discharge curves of Li–S batteries with graphite anode at 0.1 C, showing the stable operation after the initialising 1 st cycle. (b, c,) Specific capacities different current densities (b) and cycling stability at a current density of 1 C (c) for Li–S batteries with graphite anode.

Building on the compatibility of graphite with QSSE, we fabricated Li–S batteries with graphite anodes. It is worth noting that the use of graphite as an anode can cause the cells to lack lithium; therefore, a pre-lithiation step is needed. Various methods have been reported, such as the spontaneous lithiation by attaching a thin lithium foil to the surface of graphite anode or sulfur cathode during the cell assembly.^24,25^ Here, in order to characterise the graphite anode in a better manner, we adopted the cathode pre-lithiation approach by fabricating lithium sulfide (Li_2_S, the final discharge product of Li–S batteries) into the cathode (see Methods). Accordingly, the cells need to be charged first following their assembly. During this process, the Li^+^ ions are released from the cathode and transported to the anode, where they can intercalate into graphite. This step is observed in the GCD curves in Fig 5a, where the charge profile for the 1 st cycle exhibits a considerable overpotential and also additional capacity due to irreversible side reactions. Thereafter, the Li–S cells using graphite anode operate stably with a specific capacity of 1063 mAh g^−1^ at 0.1 C (1 C= 1672 mAh g^−1^), comparable to that of 1146 mAh g^−1^ achieved by the cells with metallic lithium anode. A closer observation of the GCD and cyclic voltammetry (CV, Figure S2) profiles shows that the second discharge plateau is slightly below 2.0 V, which is lower than the typical value of   ~2.1 V commonly seen in the Li–S batteries. This is in agreement with the standard electrode potential of graphite (~0.1 V *vs*. Li^+^/Li), resulting in a reduction in the overall nominal voltage of the cells.

Our previous study reported that by using QSSE in Li–S batteries (lithium foil as anode), it is possible to achieve ~ 80% capacity retention after 200 cycles.^19^ To evaluate the potential real-world feasibility of the Li–S batteries with graphite anode, rate capability and cycling stability were measured. As shown in Fig 5b, even at the relative high C-rates of 1 C and 2 C, the Li–S batteries retain 57% (604 mAh g^−1^) and 44% (469 mAh g^−1^) of the initial capacity, respectively. At 1 C, the batteries retain 90.3% of original capacity after 200 cycles with Coulombic efficiency > 98% (Fig 5c). Such a cycle life, together with the improved safety and reduced cost, provides great potential for QSSE in practical Li–S batteries.

## Conclusions

In summary, we describe QSSE to address the long-standing challenge of using graphite anode in Li–S batteries with ether-based electrolytes, achieving notable improvements in cycling stability and safety. Through *in-situ* polymerisation of DOL with metallic 1 T MoS_2_ sulfur host material, the formed QSSE mitigates the problem of solvent co-intercalation with Li^+^ ions, which typically destroys the graphite structure during operation. The QSSE possesses ionic conductivity comparable to conventional LEs. Therefore, the resultant Li–S batteries deliver high capacity (~ 1200 mAh g^−1^) and superior cycling stability, demonstrating > 90% capacity retention after 200 cycles. Furthermore, the QSSE is capable of extending the electrochemical window by over 1 V and is also flame retarding, making it a promising electrolyte for safer and long-lasting Li–S battery applications. Our design enables the stable operation of graphite in ether-based systems, thereby establishing it as a robust anode material for next-generation Li–S batteries.

## Methods

### Preparation of electrodes

The cathodes consist of 1 T MoS_2_ host and Li_2_S active material. 1 T MoS_2_ was synthesised by chemical exfoliation of 2H MoS_2_ powder with organolithium as previously reported.^26–28^ Briefly, bulk 2H MoS_2_ (0.3 g) was immersed in hexane (15 mL), followed by adding n-butyllithium solution (1.6 M in hexane, 3 mL) and refluxing for 2 days under argon protection. Then, the product was washed with hexane (50 mL) for 3 times and dispersed in deionised water with the aid of ultrasonication (20 min). After centrifugation to remove the unreacted parts and residues, the resultant powder was freeze-dried to yield 1 T MoS_2_. Li_2_S composites were prepared inside an argon-fill glovebox by mixing 1 T MoS_2_ host material (40 mg) and Li_2_S powder (100 mg) through a ball milling process. The mixture was next ground with poly (vinylidene fluoride) binder at a mass ratio of 9:1 in N-methyl-2-pyrrolidone to form a homogeneous slurry. The slurry was then coated onto carbon-coated Al foils using a doctor blade and dried at 60 °C for 12 h under vacuum. The anode slurry was prepared by mixing graphite, Super P carbon and poly(vinylidene fluoride) binder at a mass ratio of 8:1:1 in N-methyl-2-pyrrolidone, followed by coating it onto Cu foils with a doctor blade and drying at 120 °C for 12 h.

### Preparation of electrolytes

All electrolytes were prepared in an argon-filled glovebox. A LiTFSI slat (1.0 M) was first dissolved in mixed DOL and DME (1:1 by volume) solvents to produce a liquid precursor. The LE was prepared by dissolving LiNO_3_ (0.2 M) in the precursor.^29–31^ The QSSE was formed in situ by dropping the liquid precursor onto 1 T MoS_2_-based cathodes during cell assembly. Note that the QSSE without integration into cells can also be obtained by adding 1 T MoS_2_ powder (~ 0.5 mg) to the liquid precursor (2 mL). Commercial carbonate electrolyte LiPF_6_ (1.0 M in EC/DEC) was used as purchased without further modification.

### Materials characterisations

Mechanical properties and structural information of materials were measured by universal tensile testing machine (Instron Corporation), thermogravimetric analyses (Setaram Setsys Evolution 18) under an argon atmosphere, Raman spectroscopy (Renishaw InVia with a 514 nm laser beam) and XRD (Bruker D8 using Cu Kα radiation). Combustion test was carried out by igniting the electrolytes with a flame.

### Electrochemical characterisations

Electrochemical performance was characterised in coin cells (CR2032) and pouch cells (6 cm × 4.5 cm in dimension). Coin cells were fabricated in an argon-filled glovebox. Li–S coin cells were assembled with the Li_2_S cathode, the graphite anode, Celgard separator and the electrolyte. Li||graphite coin cells were assembled with similar components but with changing the electrodes to lithium foil and graphite. Pouch cells were fabricated in a dry room (relative humidity < 0.1%) with the Li_2_S cathode (Al current collector), the graphite anode (Cu current collector), Celgard separator and the electrolyte. The Al and Ni tabs were welded together with the cathodes and anodes, respectively. The entire cell core was encapsulated in the Al-laminated films. A detailed weight breakdown of the pouch cells is provided in Table S1. An external pressure of 130 kPa was applied to the pouch cells during measurements using a uniaxial pressure setup.

GCD tests were carried out on a battery cycler (LANHECT3002A) in the voltage range of 1.6 to 2.8 V at various C-rates for Li–S cells and 0 to 2.5 V for Li||graphite cells. The initial cycle of Li–S cells was charged to 3.5 V to fully active the cathode. CV and LSV tests were conducted with an electrochemical workstation (BioLogic VSP-300) at a scan rate of 0.1 mV s^−1^. Electrochemical impedance spectroscopy was measured at open circuit under a sinusoidal signal over the frequency range from 100 kHz to 100 mHz with an amplitude of 10 mV. An oven and a fridge were coupled to the battery cycler to control the temperature during the measurements.

## Supplementary Information

Below is the link to the electronic supplementary material.

Supplementary file1 (DOCX 199 KB)

## Data Availability

All data supporting the findings of this study are available within the paper.

## References

[1] M. Zhao, B.Q. Li, X.Q. Zhang, J.Q. Huang, Q. Zhang, ACS Cent. Sci. **6**(7), 1095–1104 (2020) 32724844 10.1021/acscentsci.0c00449 PMC7379100

[2] Z.X. Chen, M. Zhao, L.P. Hou, X.Q. Zhang, B.Q. Li, J.Q. Huang, Adv. Mater. **34**, 2201555 (2022) 10.1002/adma.202201555 35475585

[3] Q. Pang, X. Liang, C.Y. Kwok, L.F. Nazar, Nat. Energy **1**, 16132 (2016)

[4] L. Shi, S.M. Bak, Z. Shadike, C. Wang, C. Niu, P. Northrup, H. Lee, A.Y. Baranovskiy, C.S. Anderson, J. Qin, S. Feng, X. Ren, D. Liu, X.Q. Yang, F. Gao, D. Lu, J. Xiao, J. Liu, Energy Environ. Sci. **13**, 3620–3632 (2020)

[5] X.B. Cheng, C. Yan, J.Q. Huang, P. Li, L. Zhu, L. Zhao, Y. Zhang, W. Zhu, S.T. Yang, Q. Zhang, Energy Storage Mater. **6**, 18–25 (2017)

[6] E. Cha, M.D. Patel, J. Park, J. Hwang, V. Prasad, K. Cho, W. Choi, Nat. Nanotechnol. **13**, 337–343 (2018) 29434261 10.1038/s41565-018-0061-y

[7] X. Song, X. Liang, J. Eko, H.H. Sun, J.M. Kim, H. Kim, Y.K. Sun, Adv. Energy Mater. **14**, 2402506 (2024)

[8] D. Lin, Y. Liu, Y. Cui, Reviving the lithium metal anode for high-energy batteries. Nat. Nanotechnol. **12**, 194–206 (2017) 28265117 10.1038/nnano.2017.16

[9] C.X. Bi, M. Zhao, L.P. Hou, Z.X. Chen, X.Q. Zhang, B.Q. Li, H. Yuan, J.Q. Huang, Adv. Sci. **9**, 2103910 (2022) 10.1002/advs.202103910 PMC8805573 34784102

[10] M. Agostini, J. Hassoun, J. Liu, M. Jeong, H. Nara, T. Momma, T. Osaka, Y.K. Sun, B. Scrosati, A.C.S. Appl, Mater. Interfaces. **6**, 10924–10928 (2014) 10.1021/am4057166 24559093

[11] J. Hassoun, B. Scrosati, Angew. Chemie - Int. Ed. **49**, 2371–2374 (2010) 10.1002/anie.200907324 20191654

[12] M. Li, J. Lu, Z. Chen, K. Amine, Adv. Mater. **30**, 1800561 (2018) 10.1002/adma.201800561 29904941

[13] F. Duffner, N. Kronemeyer, J. Tübke, J. Leker, M. Winter, R. Schmuch, Nat. Energy **6**, 123–134 (2021)

[14] D. Lv, P. Yan, Y. Shao, Q. Li, S. Ferrara, H. Pan, G.L. Graff, B. Polzin, C. Wang, J.G. Zhang, J. Liu, J. Xiao, Chem. Commun. **51**, 13454–13457 (2015) 10.1039/c5cc05171a 26214797

[15] Y. Yu, J. Xu, K. Duanmu, V. Shutthanandan, S. Wi, Z. Yang, Y. Liu, X. Lyu, K. Qian, M. Agarwal, Z. Zhang, Y. Zhang, T. Li, C. Liu, V. Murugesan, J. Xie, ACS Energy Lett. **9**, 5002–5011 (2024)

[16] J. Gao, M.A. Lowe, Y. Kiya, H.D. Abruña, J. Phys. Chem. C **115**, 25132–25137 (2011)

[17] L.L. Jiang, C. Yan, Y.X. Yao, W. Cai, J.Q. Huang, Q. Zhang, Angew. Chemie - Int. Ed. **60**, 3402–3406 (2021) 10.1002/anie.202009738 33107707

[18] M. Chen, M. Shao, J. Jin, L. Cui, H. Tu, X. Fu, Energy Storage Mater. **47**, 629–648 (2022)

[19] Z. Li, Z.J. Yang, J. Moloney, C.P. Yu, M. Chhowalla, ACS Nano **18**, 16041–16050 (2024) 38833631 10.1021/acsnano.4c05002 PMC11191740

[20] Z. Lu, L. Yang, Y. Guo, J. Power. Sources **156**, 555–559 (2006)

[21] M. Shaibani, M.S. Mirshekarloo, R. Singh, C.D. Easton, M.D. Cooray, N. Eshraghi, T. Abendroth, S. Dörfler, H. Althues, S. Kaskel, A.F. Hollenkamp, Sci. Adv. **6**(1), eaay2757 (2020) 31922008 10.1126/sciadv.aay2757 PMC6941919

[22] Q. Zhao, X. Liu, S. Stalin, K. Khan, L.A. Archer, Nat. Energy **4**, 365–373 (2019)

[23] A.M. Stephan, Eur. Polym. J. **42**, 21–42 (2006)

[24] F. Wang, B. Wang, J. Li, B. Wang, Y. Zhou, D. Wang, H. Liu, S. Dou, ACS Nano **15**, 2197–2218 (2021) 33570903 10.1021/acsnano.0c10664

[25] H. Zhang, Y. Zhang, C. Cao, W. Zhao, K. Huang, Y. Zhang, Y. Shen, Z. Li, Y. Huang, Energy Environ. Sci. **17**, 7047–7057 (2024)

[26] G. Eda, H. Yamaguchi, D. Voiry, T. Fujita, M. Chen, M. Chhowalla, Nano Lett. **11**, 5111–5116 (2011) 22035145 10.1021/nl201874w

[27] Z. Li, I. Sami, J. Yang, J. Li, R.V. Kumar, M. Chhowalla, Nat. Energy **8**, 84–93 (2023)

[28] Z.J. Yang, Z. Li, G.I. Lampronti, J.I. Lee, Y. Wang, J. Day, M. Chhowalla, Chem. Mater. **36**, 4829–4837 (2024)

[29] S.S. Zhang, Electrochim. Acta **70**, 344–348 (2012)

[30] D. Aurbach, E. Pollak, R. Elazari, G. Salitra, C.S. Kelley, J. Affinito, J. Electrochem. Soc. **156**, A694–A702 (2009)

[31] Z. Li, M. Chhowalla, Nat. Chem. Eng. **1**, 563–564 (2024)

