# Supplementary material for: Stabilising graphite anode with quasi-solid-state electrolyte for long-life lithium–sulfur batteries
Source: MRS Energy Sustain. 2025 Jul 14;12(2):262–9. doi: 10.1557/s43581-025-00139-0 (PMC12572067; doi:10.1557/s43581-025-00139-0)
Supplement: Supplementary file 1 — Supplementary file1 (DOCX 199 KB) [file 43581_2025_139_MOESM1_ESM.docx]

Supporting information

**Stabilising graphite anode with quasi-solid-state electrolyte for long-life lithium-sulfur batteries**

*Zhuangnan Li^1^, Ziwei Jeffrey Yang^1^, and Manish Chhowalla^1^**

^1^Department of Materials Science and Metallurgy, University of Cambridge, Cambridge, UK

^*^email: [mc209@cam.ac.uk](mailto:mc209@cam.ac.uk)


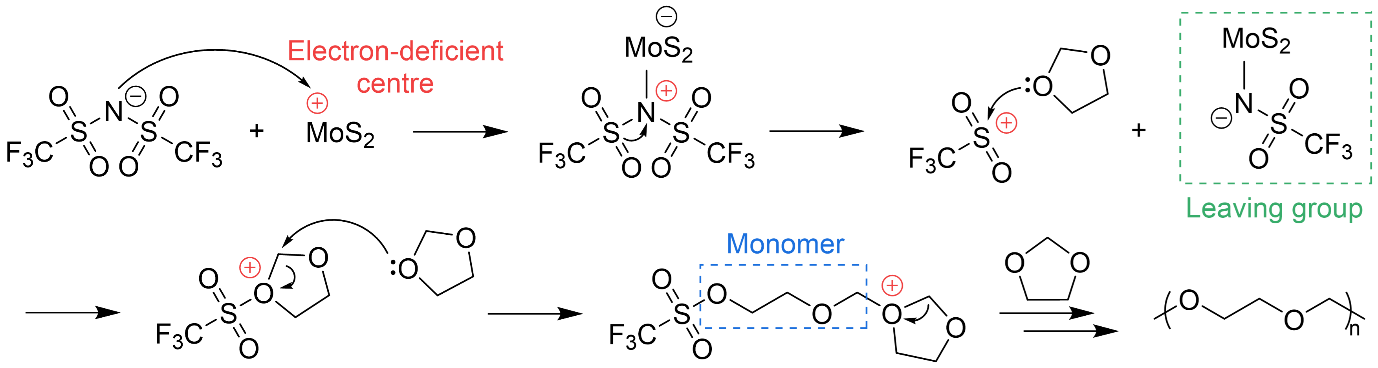


**Figure S1.** Reaction mechanism illustrating the  preparation of QSSE through the ring-opening polymerization initiated by MoS_2_-based cathodes.


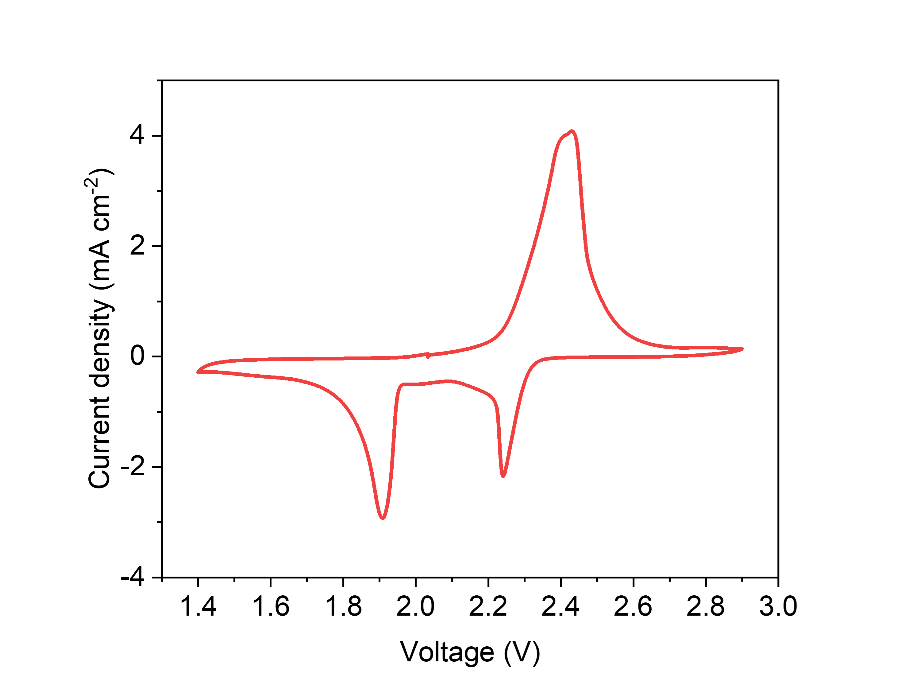


**Figure S2.** Cyclic voltammetry curves of Li−S batteries with graphite anode at a scan rate of 0.1 mVs^-1^, suggesting the typical dissolution-based stepwise reaction pathway after the initialising 1st cycle.

**Table R1.** Components mass of Li-S the pouch cell with graphite anode.

| Component | Cathode | Anode | Electrolyte | Separator | Al foil | Cu foil | Total |
| --- | --- | --- | --- | --- | --- | --- | --- |
| Mass (mg) | 110 | 310 | 486 | 36 | 55 | 145 | 1142 |
